# Supplementary material for: Adaptive metabolic reprogramming of the brain tissue during late-stage Trypanosoma brucei infection maintains host learning and memory
Source: PLoS Pathog. 2026 Oct 22;22(10):e1014734. doi: 10.1371/journal.ppat.1014734 (PMC13719033; doi:10.1371/journal.ppat.1014734)
Supplement: Supplementary file 1 [file ppat.1014734.s015.docx]

Prof Michael D Urbaniak, FRSB

Biomedical & Life Sciences

Lancaster University

Bailrigg

Lancaster LA1 4YG

+44 (0)1524 593104

[m.urbaniak@lancaster.ac.uk](mailto:m.urbaniak@lancaster.ac.uk)

Dear Editors,

**R.E. Adaptive metabolic reprogramming of the brain tissue during late-stage Trypanosoma brucei infection maintains host learning and memory (PPATHOGENS-D-26-01185)**

Please find attached a resubmission of the above research article for your consideration for publication in *PLoS Pathogens.* We would like to thank the reviewers and editors for their thorough reviews and helpful suggestions to improve the manuscript.

Below we have provided a detailed list of responses to the editors and reviewers comments, and a description of the changes that we have made to the manuscript. Attached are two versions of the manuscript, one with tracked changes, and the other a clean version. We can confirm that figures have been uploaded to PACE to ensure they meet PLoS requirements.

Best wishes,

Mick Urbaniak

------------------------------------------------------------------------------------------------------------------------

**Additional Editor Comments:**
This manuscript was deemed to be of interest to the field and the study was felt to be well-performed. However there are several points which need to be amended or clarified before this manuscript can be considered for publication in PLOS Pathogens.

Please address all of the comments raised by the three reviewers (detailed below) and also please acknowledge more fully the prior work on tryptophan metabolism in Human African Trypanosomiasis (e.g Sternberg et al., J Infect Dis. 2017 215:806-812. doi: 10.1093/infdis/jiw623).

*Response* – *We thank the editor for this suggestion. This reference has been included in the manuscript as [12], as we agree it is relevant and should have been cited.*

**Journal Requirements:**

If the reviewer comments include a recommendation to cite specific previously published works, please review and evaluate these publications to determine whether they are relevant and should be cited. There is no requirement to cite these works unless the editor has indicated otherwise.

1) We ask that a manuscript source file is provided at Revision. Please upload your manuscript file as a .doc, .docx, .rtf or .tex.

*Response – The manuscript has been uploaded as a .doc file*

2) We noticed that you used the phrase 'data not shown' in the manuscript. We do not allow these references, as the PLOS data access policy requires that all data be either published with the manuscript or made available in a publicly accessible database. Please amend the supplementary material to include the referenced data or remove the references.

*Response – We cannot find the use of the phrase ‘data not shown’ in the manuscript or supplementary files by manual inspection and text-based searching, and are happy to provide all data if you can indicate where the phrase has been accidently included.*

3) We do not publish any copyright or trademark symbols that usually accompany proprietary names, eg ©,  ®, or TM  (e.g. next to drug or reagent names). Therefore please remove all instances of trademark/copyright symbols throughout the text, including:
- TM on pages: 25, and 28.

*Response – TM removed*

4) Please upload all main figures as separate Figure files in .tif or .eps format. For more information about how to convert and format your figure files please see our guidelines: 
<https://journals.plos.org/plospathogens/s/figures>

*Response – Figures uploaded in .tif format*

5) Please upload a copy of Figure Fig 1-8 which you refer to in your text on pages 5, 7, 9, 11, 12, 14, and 18. Or, if the figure is no longer to be included as part of the submission please remove all reference to it within the text.

*Response – Figures 1-8 uploaded in .tif format. Fig 1 modified to ensure compliance with copyright.*

6) Please ensure that all Figure files have corresponding citations and legends within the manuscript. Currently, Fig 6 in your submission file inventory does not have an in-text citation. If the figure is no longer to be included as part of the submission, please remove it from the file inventory.

*Response – An in-text citation has been added for Fig 6; it was mistakenly labelled as Fig 7 in the original submission*

7) Thank you for stating "RNAseq data has been deposited with the Sequence Read Archive entry PRJNA1366789" However, we were unable to access the sequence data using the information provided. Please review and update the Data Availability Statement to ensure that the underlying data are publicly accessible and that all accession numbers, links, or repository details are correct and functional.

*Response – Apologies, the deposit has been made publicly accessible on SRA.*

**Major Issues: Key Experiments Required for Acceptance**

**Reviewer #1**: N/A

**Reviewer #2**: Tryptophan metabolism turn over in the brain by these parasites has been known for years , including the parasite ‘s deamination enzymes and more involved in this metabolism. The authors seem to write this in the abstract as though they have discovered this for the first time?

*Response: We do not claim that parasite induced brain tryptophan turnover is novel in the abstract, we only state that our model reproduces clinical observations. We now note that the clinical observation is well established.*

*L23: “The murine HAT model reproduces the decrease in brain tryptophan well established in clinical samples”.*

*We also reference the previous work in the area extensively (L84 – L95; references [10 – 19]) including the transamination reaction:*

*L87: “T. brucei cultured in vitro significantly deplete tryptophan from the media [13] which they use in both protein synthesis and transamination [14], secreting the unused biochemical intermediates of the transamination reactions as aromatic keto-acids [10, 15, 16].”*

Can the authors be a bit more specific with their day of collection of tissues from the mice, a range of a week for each S1 ES2 and LS2 is not sufficient

*Response: We have reviewed our manuscript to ensure that this is clearly communicated. The exact timing of tissue collection (all day 7, 14 or 22) is contained within the figure legends where the relevant data is presented, and a schematic overview of the experimental regimen is also shown in S1Figure.*

So Figure 1 clearly shows tissue distribution within the time frames, this is not novel, but is important control to show the control for later analyses. However, the brain signal is weak or non-existent, there is signal in stage 1 in fig 1B, so unsure of the corresponding values in figure 1C. What control was used to allow comparisons across different animals on different days.

*Response: We agree that the bioluminescent parasite signal from brain is weak, particularly at stage I of infection when it is expected that few parasites are found within the brain. We also recognise that even while levels are higher in the brain during stage II, levels are much lower in brain than in other tissues, and we specifically make this point in the manuscript.*

*L133 “Bioluminescence intensity within the brain was significantly lower (p < 0.05) than that in other tissues (Figure 1B and C).”*

*Figure 1B is a false-coloured luminescence image, we selected our images in this figure to be typical of each time point, and show the images from all infected animals in the Supplementary S2-5 Figures, so readers can visually inspect all of the raw data. Figure 1C is the quantified data showing interquartile range (box), range (whiskers) and mean (X). No control is required to allow comparison between different days of infection as absolute luminescence was recorded on a calibrated instrument.*

Fig 2A supersedes Fig 1, in terms of showing parasite burden in the brain, so why are the luminescent pictures so different between these two figures.

*Response: We apologise for any confusion. In Figure 2A the scale of the false-coloured luminescence brain images has been adjusted to allow the reader clearer visualisation of the brain. The luminescence intensity shown in Fig 1C & 2B are identical data, and are included in Fig. 2B for ease of comparison with HPLC data.*

*To remove any potential for confusion we have added values to the colour key in each figure and altered the legend of Fig 2 to explicitly state that the colour scale has changed (L163):*

*“data as shown in Fig 1 with but with an altered false-colour scale to allow visualisation of parasite localisation at lower burden.”*

Can the authors explain why in the infected S1 Fig 2B tryptophan levels seem to be so much higher.

*Response: This was unexpected particularly as other acute infections are largely associated with a reduction in tryptophan levels, partly driven by pro-inflammatory cytokines. We are currently unsure of the mechanism involved during S1 trypanosome infection that contribute to this effect. However, our observation is consistent with clinical data (Ref [11]) as stated in the discussion (L389), supporting the translational relevance of this observation.*

The tryptophan levels the authors detect in the brain is also done by LC-MS, showing a ~30% decrease, so was this decrease also observed in the blood?

*Response: We did not measure tryptophan levels in blood. Published data reports reduced blood tryptophan levels in trypanosome infection (Newport et al (1977), J. Parasitology, PMID: 321737).*

Tryptophan levels in blood and the relative turnover of it in the blood versus the brain, where there is still ample blood supply, so how do the authors distinguish these two pools. You have done this for other organs but not blood.

*Response: We quantified tryptophan levels in the brain (Fig 2) and selected peripheral tissues - Liver, Muscle and Spleen (Fig 3) and do not see a consistent trend in tissue tryptophan levels across the infection and tissue type that would simply correlate with changes in tryptophan availability in the blood. Thus, our data provide important new insights into tissue tryptophan levels across the time course of infection.*

*Unfortunately, given the high level of circulation present within the brain, and the other tissues we have measured (e.g. liver, muscle) currently available technologies do not allow us to parse out tryptophan contributions from the innervating vasculature (blood) versus the brain tissue itself.*

Unsure what the “lower” 3 panels of fig 3 are showing the parasite burden again, but this is not clearly described in the legend, again a repeat of data from fig 2.

*Response: We apologise for the lack of clarity. The colour scheme and figure legend of Fig 2 & 3 have been updated to improve clarity, with all control samples in blue and all infected samples shown in red for consistency. The data displayed in Fig 2 & 3 are for different tissues.*

The authors use of 14C 2-deoxy-glucose (2-DG) to show glucose utilisation, but 2-DG inhibits glycolysis? Despite this the control and infected images do tell a story, however the quantification of the signal is seems to vary hugely compared to those found in the lit and especially those in ref 22 , on which the method is based.

*Response: We thank the reviewer for highlighting these important points. 2-deoxy-glucose can be transported into cells, phosphorylated by hexokinase to 2-deoxy-D-glucose-6-phosphate trapping it in the cytosol but this cannot enter the glycolytic pathway as it cannot be processed by Phosphoglucose Isomerase (PGI). At high concentrations 2DG acts a competitive inhibitor of glycolysis by out competing glucose at hexokinase, but we use trace amounts of radiolabelled 2DG over a short period of time, ensuring that the levels used are not sufficient to compete with glucose at the hexokinase enzyme. The method used was developed by Sokoloff et al., 1977 (J Neurochem 28:897-916), does not significantly inhibit glycolysis, and has been extensively used to measure glucose turnover in the brain and other tissues.*

*In reference [22] brain region metabolism is normalised to the whole brain average, or that of a specified brain region (which we showed to induce errors in interpretation), generating the 14C-2-DG uptake ratio. This reduces the variability of the data, as it controls for variations in brain tissue exposure to 14C-2-DG levels that can occur across animals, for example through small alterations in the injection site. However, in the context of this study, this normalisation step is not appropriate, as there is a global decrease in 14C-2-DG levels in infected animals. Thus, if we normalised to the whole brain average 14C-2-DG levels, some regional reductions in 14C-2-DG uptake would be lost. In such circumstances, reporting the raw 14C-2-DG utilisation data (pmol/g/min), as in our study, is the most appropriate. Despite the resulting increase in data variability, it is clear from our data (as indicated in the boxplots) that stage 2 infection results in a biologically meaningful and significant reduction in both global (whole brain average) and regional glucose utilisation.*

Could the glucose depletion not be just due to the parasites utilising all of it ?

*Response: We found no significant difference in plasma ^14^C-2-DG levels between control and infected groups (L213) indicating the parasite presence is not depleting circulating levels of the tracer. If brain-resident parasites utilised significant quantities of ^14^C-2-DG we would actually see an increase in ^14^C signal within infected brains compared to controls, the opposite of the effect that we report. This strongly suggests that the changes in glucose utilisation that we see are driven by alterations in brain metabolism (as also supported by our RNAseq data) rather than by the increased parasite burden seen in brain tissues during S2 infection. We have noted this in our manuscript (L207):*

*“T. brucei rapidly consumes glucose and can take up 2-DG [23] and therefore parasite presence in the cerebral tissue could increase the apparent rate of glucose utilisation within the infected brains. However, this is contrary to our observation of decreased 2-DG utilisation.”*

The learning and memory tests of the LS2 infected mice were not affected, which is surprising given the claim parasite load and potential change in brain biochemistry , including glucose and tryptophan metabolism.

*Response: We agree that this is highly surprising (L410), and we have presented extensive evidence to back up our interpretation that adaptive metabolic reprogramming occurs in the brain tissue during infection to maintain host learning and memory. We also discuss this extensively in our discussion section.*

The authors used RNAseq to assess the changes of the host’s gene expression, showing that insulin II availability, however claiming that the expression of mitochondrial encoded genes, means the number of mitochondria are significantly unaffected is far too speculative. However they go on to claim “Overall, these data support dynamic, adaptive regulation of mitochondrial metabolism across the infection time course,”.. which is counter to their previous claim.

*Response: We thank the reviewer for highlighting this point. There is an important distinction here that requires further consideration. Our statement is that (L310)*

*“the expression of microchondrial encoded genes is not significantly altered in the brain tissue”.*

*As these genes are encoded on the mitochondrial genome, this lack of an effect may be taken as an indication that total mitochondrion numbers, as regulated by mitochondrial biogenesis and degradation, are not altered in the brains of infected animals. We do recognise that the suggestion that interpreting mitochondrially encoded gene as indicators of changes in mitochondrial number has limitations. It could be the case, for example, that mitochondrial numbers decreased but the expression of mitochondrial encoded genes themselves are increased. However, increased mitochondrial gene expression is dependent on the expression of mitochondrial transcription factor A (Tfam) and Mitochondrial RNA polymerase (Polrmt) and we found no evidence that these genes were expressed at higher levels in the brains of infected animals.*

*By contrast, the expression of several genes that are involved in regulating mitochondrial metabolism that are encoded in the nuclear DNA, are shown to be altered in the brains of infected mice, which may indicate that the mitochondrial metabolic processes are disrupted.*

*We have clarified the wording of this section of the manuscript (L307-311).*

*“Intriguingly, in both stages of infection, the expression of mitochondrial encoded genes was not significantly altered in comparison to naïve animals, and neither were the mitochondrial transcription factor A (Tfam) and Mitochondrial RNA polymerase (Polrmt) transcripts that control mitochondrial encoded gene expression. Taken together this suggests that mitochondria number were not significantly altered in the brain tissue. In contrast, the expression of several nuclear encoded mitochondrial genes known to regulate mitochondrial metabolic function were significantly increased in S1 brains.”*

The authors claim that increased lipolysis will result due to lack of glycolysis , so have the authors seen a decrease in the hormone adiponectin, which regulates lipolysis.

*Response: We did not detect any significant expression of adiponectin (Adipoq) in the brain in niave or infected mice (average FKPM < 1), as would be expected in whole brain tissue. We do detect transcripts for the Adiponectin receptors (AdipoR1, AdipoR2) but they are not significantly altered over the course of the infection. Instead, we propose the reduced brain insulin II (Ins2) is responsible for increased lipolysis (L325).*

Again the authors suggest that increase of APOL1 homologue is a direct response to the Trypanosoma infection, how would the mouse know this in a way to clear the brain of the parasites?

*Response: Human APOL1 expression is induced by pro-inflammatory cytokines (Toole et al., (2018), PMID: 29110761) and is key to the lysis of non-human infective trypanosome species (Ref [30]). More generally the murine apolipoprotein family of gene are strongly induced by inflammation and driven by pro-inflammatory cytokines, i.e. Apolipoprotein L9 isoforms ApoL9a and ApoL9b expression is directly stimulated by IFN (L333, Ref [31]). Thus, the highly pro-inflammatory environment induced by trypanosome infection seen in our RNAseq data is likely a key driver of the expression of these genes during trypanosome infection in mice.*

The very low percentage of reads for the parasites RNAseq is not robust enough to make any meaningful conclusions.

*Response: The low percentage of reads mapping to the trypanosome genome is entirely expected from RNAseq analysis of infected tissue, as the host cells vastly outnumber the parasite cells. We are confident that the quality of reads (Q20 > 95%) and depth of reads (~6Gb per sample) provided acceptable coverage of the parasite transcriptome with 6379 protein-coding transcripts detected with an average FKPM ranging from 7.4 – 64.3 across replicates. Furthermore, the identification of DEGs was made using DEseq2, which takes into account read depth and replicate variability, using the same cut-off values (|log2(FC)|> 1 & padj < 0.05) as the host data and is therefore statistically rigorous.*

*We acknowledge that genes with low expression levels will not be captured in this analysis, and have modified the manuscript to clarify this point (L335):*

*“Since host cells vastly outnumber parasite cells in the tissue only a low percentage of reads from the S1 and ES2 samples map to the T. brucei genome (0.03 – 0.18%), limiting the detection of genes with low expression levels. Despite this 6,378 protein-coding transcripts (S5 Table) were identified from the 10,239 annotated T. brucei genes with a moderate but significant correlation between biological replicates (Pearson correlation 0.62 – 0.84).”*

Very surprised that more was made from the metabolic data?

*Response: Apologies, we are not sure what this question is asking (is there a missing “not”?). We have presented, interpreted and discussed the metabolic data, and made conclusions that are backed by the evidence we present.*

Can the authors please provide a statement on the statistical analysis used.

*Response: We thank the reviewer for highlighting this. We have reviewed our reporting of the statistical analysis approaches we have used. For most of our output measures this is reported at the end of the relevant methods section. We now also highlight subsections in the methods where statistical analysis is mentioned in the subsection title, where appropriate (e.g. behavioural video analysis section). We note that we had missed reporting of the statistical analysis 2-DG brain image analysis in the relevant methods section. This is now included in the relevant section (L629) and is also mentioned in the legend of Fig 4 (L224).*

The authors may want to consider the parallels between the brain’s switch to fatty acid as a carbon source in stage II HAT and that in an ageing brain, which is exaggerated in Alzheimer’s disease.

*Response: Whilst Alzheimer’s disease is characterised by a reduction in brain glucose metabolism, and the potential neuroprotective role of ketone bodies in enhancing brain metabolism is an emerging area of therapeutic interest, the reduced brain glucose metabolism in Alzheimer's disease is caused by neurodegeneration and synapse loss that is not seen in HAT. Therefore, we are aiming to be very cautious in extrapolating our observations, which are induced by infection by a specific brain-resident parasite, and those that result from multi-decade neurodegenerative disease such as Alzheimer’s. While there is also evidence of the fatty acid switch in the aged brain, as noted by the reviewer, we also want to be cautious around suggesting the relevance of our typanosome-induced changes to those seen in normal ageing. Therefore, to ensure that we are not over-interpreting the relevance of our data to very different biological phenomenon, we have not included discussion around these points in our manuscript.*

**Reviewer #3:** No additional experiments. However, some consideration on interpretation.

1. In the Bioluminescent model, it seems diminazene or another stage 1 drug wasn't used to clear parasites from blood to focus on parasites in brain. Was this to assure a natural progression i.e. retaining blood and CNS parasites to have a more "natural" response?

*Response: Yes, the reviewer is correct in their interpretation. Our focus was on characterising the systemic and brain changes that occur only in response to infection, rather than trying to recapitulate complex clinical scenarios that include confounds, such as stage 1 drug administration, that would introduce additional variables into our observations. Our study represents a first-stage in understanding the events that occur only during natural infection.*

2. Distribution. Did you look at skin? I presume not as its relatively difficult to obtain, but given the interest in skin tropism it would have been of interest.

*Response: We did collect skin samples for bioluminescence analysis at ES2 and LS2 (Fig 1B & C), but due to an omission in sample collection have no data for S1. The raw bioluminescent images showing skin samples can be seen in Figure 1 and supplemental figures S2-S5. Skin samples for ES2 and LS2 animals were included in the bottom right well on the images. There is little apparent tropism during ES2, however clear bioluminescence of skin samples can be seen in LS2 infected animals to varying degrees.*

3. Tryptophan measurements by two methods consistent with each other and apparently not related to trypanosome numbers (although trypanosomes are prodigious consumers of tryptophan. Could it be that replenishment after trypanosome consumption is more difficult in the CSF?) It is a pity tryptophan metabolites weren't sought as it would be interesting to know what is happening to indole pyruvate (trypanosome product) versus host tryptophan metabolic products (serotonin, kynurenine pathway). However, following tryptophan alone is already of interest, particularly in having looked at different organs across the duration of infection.

*Response: We do not see consistent trend in tissue tryptophan levels across the infection and tissue type that would simply correlate with changes in tryptophan availability in the blood, but do appear to correlate with previously reported changes in tryptophan availability in the CSF (Ref [11]). Even though trypanosomes are prodigious consumers of tryptophan (Ref [10 - 13]) we see increased tryptophan in peripheral tissues at the higher parasite burden of LS2 when the level in the brain is reduced.*

*We agree that quantifying host and parasite derived tryptophan metabolites would be of interest, particularly as Ref [11] reports that during stage II infection CSF has reduced tryptophan and increased levels of metabolites on both the serotonin (5-hydroxytryptophan) and kynurenine pathways (kynurenine) but failed to detect indole 3-pyruvate.*

4. Glucose utilisation. It seems to me that the data are more consistent with the loss of glucose (and 2-DOG) uptake into the brain as all compartments had diminished levels. If glucose uptake into the brain were not affected then decreased glucose utilisation would correlate with increased accumulation (although 2-DOG is only metabolised as far as the 6-phospho derivative hence would accumulate as that metabolite - so it isn't actually utilised per se). Although transcriptomics showed no change in transcript levels of glucose transporter genes, post-transcriptional regulation is more likely, especially in am insulin response where glucose transporter trafficking to the cell membrane is influenced. This needs to be clarified.

*Response: We thank the reviewer for raising these interesting points. As mentioned, we have no transcriptional evidence to support a decrease in glucose transport into the brain during S2 infection, but agree that we cannot exclude the possibility that post-transcriptional regulation of glucose transporter activity occurs. As the outcome of reduced glucose transport into the brain will be reduced glucose metabolism this alternative explanation (for which we have no evidence) would not alter our conclusion that adaptive metabolic reprogramming of the brain occurs to maintains host learning and memory.*

*We agree that the decreased insulin II expression we have identified may act to reduce tissue glucose uptake by downregulating Glut4 glucose transporter activity in insulin-sensitive cells through post-transcriptional mechanisms to reduce uptake of glucose. However, as cerebral tissue relies on the insulin-insensitive Glut1 and Glut3 glucose transporters for the majority of their glucose uptake, the effect of reduced Glut4 activity is likely insufficient to explain the 1.8-fold global reduction in ^14^C-2-DG we observe.*

*To clarify these points we have re-organised this section of the manuscript and included extra details on the potential impact of post-transcriptional regulation (L297):*

*“There was no evidence that glucose transporter gene expression (Slc2a1 – Slc2a5) was significantly altered in ES2, suggesting that glucose transport in the CNS was not transcriptionally regulated during infection, although we cannot exclude the possibility of post-transcriptional regulation of glucose transporter activity. Consistent with the reduced cerebral glucose metabolism we identified in LS2 mice, expression levels of the Insulin II (Ins2) gene, which regulates cerebral tissue glucose use [28], was dramatically decreased by 18-fold in ES2 brains (Fig 7). We saw no significant change in ES2 mice in the expression of genes in the GO cellular response pathways for insulin signalling (GO:1900076) and the regulation of glucose import in response to insulin stimulus (GO:2001273), suggesting that the reduced level of Insulin II will have functional consequences that are not confounded by transcriptional alterations in these GO pathways. Reduced Insulin II availability could downregulate Glut4 glucose transporter activity in insulin-sensitive cerebral cells through post-translational mechanisms, reducing the uptake of both glucose and ^14^C-2-DG. However, as the majority of glucose uptake by cerebral tissue relies on the insulin-insensitive Glut1 and Glut3 glucose transporters, any effect of reduced Glut4 activity is likely insufficient to explain the 1.8-fold global reduction in cerebral glucose metabolism we observed in LS2.”*

5. Reduced glucose utilisation would be unusual in any case in a pro-inflammatory state where in most immune cell types glucose utilisation is upregulated. One wonders, therefore, whether this study shows an interesting phenomenon, i.e. does the brain respond to inflammatory triggers by diminishing uptake of glucose, with a functional requirement to curtail the pro-inlammatory state to some extent because of the pathogenic risks of inflammation in the brain?

*Response: We thank the reviewer for this interesting suggestion and agree that this was a surprising finding, having expected to see increased glucose use coupled to the inflammatory response.*

6. Behavioural changes in mice. This is one of the extremely rare examples where mouse behaviour in response to trypanosome infection of the brain has been measured. I could only find one other study and because of that scarcity I think some reference to that work is required (and comparisons to finding here). PMID:40977748

*Response*: *We agree that the paper is relevant and have now included it in the manuscript as ref [35] and modified the discussion section accordingly:*

*L412 “LS2 was accompanied by a reduction in locomotion in the OFT, although surprisingly we did not observe any alteration in episodic learning or memory in NORT despite the profound changes in brain glucose utilisation and tryptophan levels observed. This is particularly unexpected given the reduced glucose metabolism identified in brain regions including the hippocampus and prefrontal cortex, regions known to contribute to effective NORT performance [33]. Moreover, the lack of an effect on anxiety-like behaviour in the OFT was surprising given the reduced glucose metabolism seen in brain structures (e.g. amygdala nuclei) involved in this process, and the observation of reduced brain tryptophan, given its established role in anxiety-like behaviour, through its influence on serotonin neurotransmission [34]. Our results contrasts with a previous study using a different murine HAT model [35] where infection resulted in reduced anxiety in the elevated maze plus test and reduced anxiety-like behaviour in the OFT, potentially due to differences in the host and parasite strains used, although in both models infection results in reduced locomotion.”*

7. In the Discussion on line 407 they refer to RNAseq data indicating that tryptophan changes are unlikely to be host driven as transcripts aren't altered in abundance, but given the fact that so much regulation of protein activity is post-transcriptional (as probably also true of the glucose transporter situation) I think this needs to be amended. Although it might be easier to replenish trypanosome consumed tryptophan in blood than in brain - hence with the available data it isn't possible to say why these levels are changing

*Response*: *We agree that our RNAseq data cannot rule out regulation of host tryptophan metabolising enzymes through i.e. post-translational modifications that affect protein activity, a limitation common to all transcriptomic analysis. We have amended the discussion to clarify this point (L402):*

*“Our RNAseq data shows that the transcript levels of tryptophan metabolising enzymes were not significantly altered during the infection, suggesting that transcriptional regulation of host brain tryptophan metabolism does not drive the observed changes in brain tryptophan. However, we cannot exclude the possibility that the activity of host tryptophan metabolising enzymes are altered over the course of the infection through changes in post-translational modifications.”*

________________________________________

**Part III – Minor Issues: Editorial and Data Presentation Modifications**

**Reviewer #1:** Points that could be considered

• Line 60: Consider ‘generally results in death’ to acknowledge occurrence of rare self-cure and evidence of trypanotolerance

*Response: we have modified the statement clarify that only Stage II is fatal without treatment:*

*L57 “In the first stage of HAT the parasite multiplies in the blood, lymph and interstitial space, which generally progresses to a second stage where the parasite enters the central nervous system (CNS) causing severe neurological disturbances that if untreated are fatal [1].”*

• Some consideration of the influence of the model / mouse strain used could be included

*Response: We have added a sentence:*

*L114 “We have used the VSL2 stain in combination with outbred CD-1 mice which display a more natural range of behaviours in comparison to in-bred mice, with infection resulting in rapid progression to S2.”*

• Lines 115-117: How were your timings demarcating the 3-infections stages defined?

*Response: These timings are based on those reported in Ref [20] which were experimentally confirmed by our bioluminescent monitoring*

• Line 118-120: Take care not to include methodology in your results.

*Response: We believe that a clear statement of the timings of experimental regimen is important for clarity, and do not provide any details of the methods used*

• Fig 1A: Why was skin not obtained for stage 1 samples?

*Response: This was a simple omission in data collection*

• Line 133-134: Fig 1C appears to show brain with similar bioluminescence to several other tissues

*Response: The observed parasite burden varied by tissue type, timepoint and individual animal, hence the use of box plots. Statistically, the brain has a significantly lower (p < 0.05) bioluminescence intensity when compared to the range of intensities measured all other tissue types; individually only the eye has comparable consistently low levels of bioluminescence.*

• Fig 1C: Was a similar weight of each tissue used for bioluminescence analysis at each time point? Eg the spleen and LN change significantly with infection. Would the size of the tissue analysed have any effect on the bioluminescence measured?

*Response: We used a similar sized piece of tissue for bioluminescence analysis and calculated intensity density from the 2D tissue outline to account for variation in sample size, but did not account for changes in tissue size. As changes in bioluminescence intensity for most tissue sampled including the spleen and mLN are >10-fold, any variation in tissue size is likely to make only a minor contribution.*

• Line 149: Expand your description of Fig 2A

*Response: The figure legend has been modified to improve clarity, and the colour scheme altered for consistency.*

• Line 155 – 159: The LC=MS confirms the results of the tryptophan analysis for LS2 animals (p< 0.05) but provides a ns result for ES2 (previously p<0.01 by HPLC). Can you explain this difference?

*Response: Different samples were subjected to analysis by HPLC and MS; higher variation in the infected ES2 samples subjected to MS results in a non-significant result which was disappointing. One possible explanation is that the infection in that individual was slower and more S1-like, although we did not find any basis to exclude the individual on unbiased criteria.*

• Line 166: you have no p<0.001 data in this figure please amend as required.

*Response: The figure legend has been modified*

• Line 170: why was ES2 not included in this analysis?

*Response: Due to cost, time constraints, and the higher variability in ES2 compared to LS2 in MS analysis we chose to focus on S1 vs LS2.*

• Line 177: remove ‘a’ after muscle

*Response: removed*

• Line 187: add ‘tested’ following tissue, to acknowledge that not all tissue types were included in the analysis

*Response: Agreed, this improves clarity*

• Pedantic I know – stick to one colour, either red or green, for the infected samples. This is red for tryptophan but green for luminescence but you only have one key.

*Response: This is a fair point to make as it will improve clarity. We have altered the colour scheme in Fig 2 & 3 so that blue indicates control and red infected; all luminescence quantitation data I snow shown in red to indicate that are from infected samples.*

• Fig 4; Can you label brain sections with broad regions corresponding to the plots

*Response: We thank the reviewer for this suggestion. We have labelled the brain sections shown for control animals with the brain regions included in the plots, where the regions measured are available in the sections shown.*

• Lines 227-243: Add specific data to text (eg mean time exploring…)

*Response: We now include direct reference to the specific data and measures taken within this section of our revised manuscript. This includes mention of distance moved (cm) as a reflection of locomotor activity, object interaction duration (secs) as a measure of task engagement during the acquisition phase of the NORT, and the percentage (%) time animals explored the novel as compared to the familiar object as a reflection of episodic learning and memory.*

• Fig 5 legend; Would be good to include total time spent in OFT

*Response: We now include, in the figure legend, information that measures in the OFT were recorded over a 15-minute period.*

• Lines 261-297: Should be Fig 6 references rather than Fig 7

*Response: corrected*

• Fig 6 legend says right hemisphere, figure 7 say left – please clarify

*Response: corrected to right*

• Why was LS2 not include in the RNAseq analysis?

*Response: Due to cost and time constraints, we prioritised characterising ES2 to record the changes characterising the development of stage II in rather than risk characterising the pathology associated with LS2.*

• Lines 414 – 417: Need to clarify the point you’re trying to make. You begin by mentioning specific areas but then describe whole brain with no further elaboration on regions

*Response: The sentence has been amended to improve clarity*. *We describe the expected effect of perturbation of particular regions in sentences that follow ( L413 -hippocampus and prefrontal cortex, L416 - amygdala nuclei).*

• Line 431: Add ‘also’ after may

*Response: Added*

• Lines 513-514: check you have included all sample types taken

*Response: Added blood and skin, and re-ordered list to be consistent with presentation in Fig 1.*

• Some of the methods seem very detailed and could be trimmed where manufactures instructions or a standard protocol are followed.

*Response: We have trimmed sections of the material and methods section as suggested, whilst retaining sufficient detail to ensure that the experiments can be reproduced.*

• Line 605: how did you take the blood sample?

*Response: The blood samples were taken post mortem from the neck via torso inversion (L607).*

• Line 607: Is this the most appropriate method to use for terminal blood sampling? What are the limitations? Contamination may be an issue.

*Response: Given the need to rapidly process the brain tissue, we feel that this is the most appropriate method to obtain a blood sample for determination of blood glucose and plasma ^14^C levels. The main limitations of this approach are that the quality of blood obtained is lower than i.e. cardiac puncture, and the sample is insufficiently sterile to be used for subsequent infections – neither of which are relevant for our intended use.*

• Line 632: Were animals perfused to remove peripheral blood prior to brain excision? If not, how may this impact your RNAseq data?

*Response: No perfusion was performed. Any peripheral blood carried over is unlikely to contain sufficient host mRNA to significantly affect the interpretation of the host RNAseq data, but we concede that given the low level of parasite RNA detected it is possible it may have an impact on the trypanosome RNAseq data interpretation.*

*Therefore, we have added this caveat to the manuscript:*

*L266 “Alternatively, as we did not perfuse brains to remove peripheral blood there may be some contamination from bloodstream-derived mRNAs.”*

*L368 “although we cannot eliminate the potentially confounding effect of contaminating bloodstream-resident parasite mRNAs.”*

**Reviewer #2:** Can the authors please provide a statement on the statistical analysis used.

*Response: See previous response above - the statistical analysis for each output measures this is reported at the end of the relevant methods section, and now including 2-DG analysis.*

Fluorescence detection of HPLC tryptophan, can the authors please add a trace or to in the supplementary data ?

*Response: A representative HPLC trace has been added to the supplementary data as S6 Figure.*

**Reviewer #3:** (No Response)
