# Supplementary material for: Adaptive metabolic reprogramming of the brain tissue during late-stage Trypanosoma brucei infection maintains host learning and memory
Source: PLoS Pathog. 2026 Oct 22;22(10):e1014734. doi: 10.1371/journal.ppat.1014734 (PMC13719033; doi:10.1371/journal.ppat.1014734)
Supplement: Peer review history

# Peer review: Decision Letter 0

**DOI:** 10.1371/journal.ppat.1014734.r001

**Authors:** Dominique Soldati-Favre, Martin Taylor

9 Jul 2026

Adaptive metabolic reprogramming of the brain tissue during late-stage Trypanosoma brucei infection maintains host learning and memory

PLOS Pathogens

Dear Dr. Urbaniak,

Thank you for submitting your manuscript to PLOS Pathogens. After careful consideration, we feel that it has merit but does not fully meet PLOS Pathogens's publication criteria as it currently stands. Therefore, we invite you to submit a revised version of the manuscript that addresses the points raised during the review process.

Please submit your revised manuscript by Sep 07 2026 11:59PM. If you will need more time than this to complete your revisions, please reply to this message or contact the journal office at plospathogens@plos.org. When you're ready to submit your revision, log on to https://www.editorialmanager.com/ppathogens/ and select the 'Submissions Needing Revision' folder to locate your manuscript file.

Please include the following items when submitting your revised manuscript:

* A letter that responds to each point raised by the editor and reviewer(s). You should upload this letter as a separate file labeled 'Response to Reviewers'. This file does not need to include responses to any formatting updates and technical items listed in the 'Journal Requirements' section below.

* A marked-up copy of your manuscript that highlights changes made to the original version. You should upload this as a separate file labeled 'Revised Manuscript with Track Changes'.

* An unmarked version of your revised paper without tracked changes. You should upload this as a separate file labeled 'Manuscript'.

We look forward to receiving your revised manuscript.

Kind regards,

Martin Craig Taylor

Guest Editor

PLOS Pathogens

Dominique Soldati-Favre

Section Editor

Editor-in-Chief

PLOS Pathogens

orcid.org/0000-0003-2946-9497

Editor-in-Chief

PLOS Pathogens

orcid.org/0000-0002-7699-2064

**Additional Editor Comments (if provided):**

This manuscript was deemed to be of interest to the field and the study was felt to be well-performed. However there are several points which need to be amended or clarified before this manuscript can be considered for publication in PLOS Pathogens.

Please address all of the comments raised by the three reviewers (detailed below) and also please acknowledge more fully the prior work on tryptophan metabolism in Human African Trypanosomiasis (e.g Sternberg et al., J Infect Dis. 2017 215:806-812. doi: 10.1093/infdis/jiw623).

**Journal Requirements:**

If the reviewer comments include a recommendation to cite specific previously published works, please review and evaluate these publications to determine whether they are relevant and should be cited. There is no requirement to cite these works unless the editor has indicated otherwise.

1) We ask that a manuscript source file is provided at Revision. Please upload your manuscript file as a .doc, .docx, .rtf or .tex. If you are providing a .tex file, please upload it under the item type u2018LaTeX Source Fileu2019 and leave your .pdf version as the item type u2018Manuscriptu2019.

2) We noticed that you used the phrase 'data not shown' in the manuscript. We do not allow these references, as the PLOS data access policy requires that all data be either published with the manuscript or made available in a publicly accessible database. Please amend the supplementary material to include the referenced data or remove the references.

3) We do not publish any copyright or trademark symbols that usually accompany proprietary names, eg ©,  ®, or TM  (e.g. next to drug or reagent names). Therefore please remove all instances of trademark/copyright symbols throughout the text, including:

- TM on pages: 25, and 28.

4) Please upload all main figures as separate Figure files in .tif or .eps format. For more information about how to convert and format your figure files please see our guidelines:

https://journals.plos.org/plospathogens/s/figures

5) Please upload a copy of Figure Fig 1-8 which you refer to in your text on pages 5, 7, 9, 11, 12, 14, and 18. Or, if the figure is no longer to be included as part of the submission please remove all reference to it within the text.

6) Please ensure that all Figure files have corresponding citations and legends within the manuscript. Currently, Fig 6 in your submission file inventory does not have an in-text citation. If the figure is no longer to be included as part of the submission, please remove it from the file inventory.

7) Thank you for stating "RNAseq data has been deposited with the Sequence Read Archive entry PRJNA1366789" However, we were unable to access the sequence data using the information provided. Please review and update the Data Availability Statement to ensure that the underlying data are publicly accessible and that all accession numbers, links, or repository details are correct and functional.

**Reviewers' Comments:**

Reviewer's Responses to Questions

**Part I - Summary**

Please use this section to discuss strengths/weaknesses of study, novelty/significance, general execution and scholarship.

Reviewer #1: Adaptive metabolic reprogramming of the brain tissue during late-stage Trypanosoma brucei infection maintains host learning and memory.

In this manuscript the authors investigate the effect of T. brucei VSL2 infection on the brain and a variety of peripheral tissues, using an established mouse model of the infection in conjunction with an appropriate range of contemporary and complementary approaches.

The authors find behavioural changes, associated with locomotion but no evidence of alterations in anxiety or memory and learning behaviours in infected animals. In contrast a wide range of metabolic and genetic alterations are demonstrated in the CNS, including a shift away from glucose metabolism towards utilisation of adipose reserves. The authors also provide ideas on how their results can be assimilated to explain their findings.

Overall the paper is well written and provides valuable insight into the complex host pathogen interactions that maintain trypanosome infections, with specific emphasis on the changes induced within the CNS of the infected host. The investigation broadens the current knowledge base in this field and should be published with minor corrections.

Reviewer #2: Tryptophan metabolism turn over in the brain by these parasites has been known for years , including the parasite ‘s deamination enzymes and more involved in this metabolism. The authors seem to write this in the abstract as though they have discovered this for the first time?

Reviewer #3: I found this to be a technically well performed study and novel in terms of metabolic and transcriptomic analysis of trypanosome infection of the mouse brain. Loss of tryptophan seen elsewhere was confirmed, but improved on previous studies looking into multiple compartments at different times during the infection. The glucose data is potentially very interesting, although I didn't agree with the authors' interpretation. Linking this molecular information to mouse behavioural studies is another key piece of novel work. Overall, several important and novel findings are reported.

**********

**Part II – Major Issues: Key Experiments Required for Acceptance**

Please use this section to detail the key new experiments or modifications of existing experiments that should be absolutely required to validate study conclusions.

Generally, there should be no more than 3 such required experiments or major modifications for a "Major Revision" recommendation. If more than 3 experiments are necessary to validate the study conclusions, then you are encouraged to recommend "Reject".

Reviewer #1: N/A

Reviewer #2: Tryptophan metabolism turn over in the brain by these parasites has been known for years , including the parasite ‘s deamination enzymes and more involved in this metabolism. The authors seem to write this in the abstract as though they have discovered this for the first time?

Can the authors be a bit more specific with their day of collection of tissues from the mice, a range of a week for each S1 ES2 and LS2 is not sufficient,

So Figure 1 clearly shows tissue distribution within the time frames, this is not novel, but is important control to show the control for later analyses. However, the brain signal is weak or non-existent, there is signal in stage 1 in fig 1B, so unsure of the corresponding values in figure 1C. What control was used to allow comparisons across different animals on different days.

Fig 2A supersedes Fig 1, in terms of showing parasite burden in the brain, so why are the luminescent pictures so different between these two figures.

Can the authors explain why in the infected S! Fig 2B tryptophan levels seem to be so much higher. The tryptophan levels the authors detect in the brain is also done by LC-MS, showing a ~30% decrease, so was this decrease also observed in the blood?

Tryptophan levels in blood and the relative turnover of it in the blood versus the brain, where there is still ample blood supply , so how do the authors distinguish these two pools. You have done this for other organs but not blood.

Unsure what the “lower” 3 panels of fig 3 are showing the parasite burden again, but this is not clearly described in the legend, again a repeat of data from fig 2.

The authors use of 14C 2-deoxy-glucose (2-DG) to show glucose utilisation, but 2-DG inhibits glycolysis? Despite this the control and infected images do tell a story, however the quantification of the signal is seems to vary hugely compared to those found in the lit and especially those in ref 22 , on which the method is based.

Could the glucose depletion not be just due to the parasites utilising all of it ?

The learning and memory tests of the LS2 infected mice were not affected, which is surprising given the claim parasite load and potential change in brain biochemistry , including glucose and tryptophan metabolism.

The authors used RNAseq to assess the changes of the host’s gene expression, showing that insulin II availability, however claiming that the expression of mitochondrial encoded genes,, means the number of mitochondria are significantly unaffected is far too speculative. However they go on to claim “Overall, these data support dynamic, adaptive regulation of mitochondrial

metabolism across the infection time course,”.. which is counter to their previous claim.

The authors claim that increased lipolysis will result due to lack of glycolysis , so have the authors seen a decrease in the hormone adiponectin, which regulates lipolysis.

Again the authors suggest that increase of APOL1 homologue is a direct response to the Trypanosoma infection, how would the mouse know this ina way to clear the brain of the parasites?

The very low percentage of reads for the parasites RNAseq is not robust enough to make any meaningful conclusions.

Very surprised that more was made from the metabolic data?

Can the authors please provide a statement on the statistical analysis used.

The authors may want to consider the parallels between the brain’s switch to fatty acid as a carbon source in stage II HAT and that in an ageing brain, which is exaggerated in Alzheimer’s disease.

Reviewer #3: No additional experiments. However, some consideration on interpretation.

1. In the Bioluminescent model, it seems diminazene or another stage 1 drug wasn't used to clear parasites from blood to focus on parasites in brain. Was this to assure a natural progression i.e. retaining blood and CNS parasites to have a more "natural" response?

2. Distribution. Did you look at skin? I presume not as its relatively difficult to obtain, but given the interest in skin tropism it would have been of interest.

3. Tryptophan measurements by two methods consistent with each other and apparently not related to trypanosome numbers (although trypanosomes are prodigious consumers of tryptophan. Could it be that replenishment after trypanosome consumption is more difficult in the CSF?) It is a pity tryptophan metabolites weren't sought as it would be interesting to know what is happening to indole pyruvate (trypanosome product) versus host tryptophan metabolic products (serotonin, kynurenine pathway). However, following tryptophan alone is already of interest, particularly in having looked at different organs across the duration of infection..

4. Glucose utilisation. It seems to me that the data are more consistent with the loss of glucose (and 2-DOG) uptake into the brain as all compartments had diminished levels. If glucose uptake into the brain were not affected then decreased glucose utilisation would correlate with increased accumulation (although 2-DOG is only metabolised as far as the 6-phospho derivative hence would accumulate as that metabolite - so it isn't actually utilised per se). Although transcriptomics showed no change in transcript levels of glucose transporter genes, post-transcriptional regulation is more likely, especially in am insulin response where glucose transporter trafficking to the cell membrane is influenced. This needs to be clarified.

5. Reduced glucose utilisation would be unusual in any case in a pro-inflammatory state where in most immune cell types glucose utilisation is upregulated. One wonders, therefore, whether this study shows an interesting phenomenon, i.e. does the brain respond to inflammatory triggers by diminishing uptake of glucose, with a functional requirement to curtail the pro-inlammatory state to some extent because of the pathogenic risks of inflammation in the brain?

6. Behavioural changes in mice. This is one of the extremely rare examples where mouse behaviour in response to trypanosome infection of the brain has been measured. I could only find one other study and because of that scarcity I think some reference to that work is required (and comparisons to finding here). PMID:40977748

7. In the Discussion on line 407 they refer to RNAseq data indicating that tryptophan changes are unlikely to be host driven as transcripts aren't altered in abundance, but given the fact that so much regulation of protein activity is post-transcriptional (as probably also true of the glucose transporter situation) I think this needs to be amended. Although it might be easier to replenish trypanosome consumed tryptophan in blood than in brain - hence with the available data it isn't possible to say why these levels are changing.\

**********

**Part III – Minor Issues: Editorial and Data Presentation Modifications**

Please use this section for editorial suggestions as well as relatively minor modifications of existing data that would enhance clarity.

Reviewer #1: Points that could be considered

• Line 60: Consider ‘generally results in death’ to acknowledge occurrence of rare self-cure and evidence of trypanotolerance

• Some consideration of the influence of the model / mouse strain used could be included

• Lines 115-117: How were your timings demarcating the 3-infections stages defined?

• Line 118-120: Take care not to include methodology in your results.

• Fig 1A: Why was skin not obtained for stage 1 samples?

• Line 133-134: Fig 1C appears to show brain with similar bioluminescence to several other tissues

• Fig 1C: Was a similar weight of each tissue used for bioluminescence analysis at each time point? Eg the spleen and LN change significantly with infection. Would the size of the tissue analysed have any effect on the bioluminescence measured?

• Line 149: Expand your description of Fig 2A

• Line 155 – 159: The LC=MS confirms the results of the tryptophan analysis for LS2 animals (p< 0.05) but provides a ns result for ES2 (previously p<0.01 by HPLC). Can you explain this difference?

• Line 166: you have no p<0.001 data in this figure please amend as required.

• Line 170: why was ES2 not included in this analysis?

• Line 177: remove ‘a’ after muscle

• Line 187: add ‘tested’ following tissue, to acknowledge that not all tissue types were included in the analysis

• Pedantic I know – stick to one colour, either red or green, for the infected samples. This is red for tryptophan but green for luminescence but yu only have one key.

• Fig 4; Can you label brain sections with broad regions corresponding to the plots

• Lines 227-243: Add specific data to text (eg mean time exploring…)

• Fig 5 legend; Would be good to include total time spent in OFT

• Lines 261-297: Should be Fig 6 references rather than Fig 7

• Fig 6 legend says right hemisphere, figure 7 say left – please clarify

• Why was LS2 not include in the RNAseq analysis?

• Lines 414 – 417: Need to clarify the point you’re trying to make. You begin by mentioning specific areas but then describe whole brain with no further elaboration on regions

• Line 431: Add ‘also’ after may

• Lines 513-514: check you have included all sample types taken

• Some of the methods seem very detailed and could be trimmed where manufactures instructions or a standard protocol are followed.

• Line 605: how did you take the blood sample?

• Line 607: Is this the most appropriate method to use for terminal blood sampling? What are the limitations? Contamination may be an issue.

• Line 632: Were animals perfused to remove peripheral blood prior to brain excision? If not, how may this impact your RNAseq data?

Reviewer #2: Can the authors please provide a statement on the statistical analysis used.

Fluorescence detection of HPLC tryptophan, can the authors please add a trace or to in the supplementary data ?

Reviewer #3: (No Response)

**********

PLOS authors have the option to publish the peer review history of their article (what does this mean?). If published, this will include your full peer review and any attached files.

If you choose “no”, your identity will remain anonymous but your review may still be made public.

**Do you want your identity to be public for this peer review?**  For information about this choice, including consent withdrawal, please see our Privacy Policy.

Reviewer #1: No

Reviewer #2: No

Reviewer #3: No

[NOTE: If reviewer comments were submitted as an attachment file, they will be attached to this email and accessible via the submission site. Please log into your account, locate the manuscript record, and check for the action link "View Attachments". If this link does not appear, there are no attachment files.]

**Figure resubmission:**

**Reproducibility:**

?>

# Author comment: Author response to Decision Letter 1

**DOI:** 10.1371/journal.ppat.1014734.r002

16 Aug 2026

Attachment Submitted filename: Response-to-reviewers.docx

# Peer review: Decision Letter 1

**DOI:** 10.1371/journal.ppat.1014734.r003

**Authors:** Tracey Lamb, Martin Taylor

13 Oct 2026

Dear Dr. Urbaniak,

We are pleased to inform that your manuscript, "Adaptive metabolic reprogramming of the brain tissue during late-stage Trypanosoma brucei infection maintains host learning and memory", has been editorially accepted for publication at PLOS Pathogens.

Before your manuscript can be formally accepted and sent to production, you will need to complete our formatting changes, which you will receive by email within a week. Please note that your manuscript will not be scheduled for publication until you have made the required changes.

IMPORTANT NOTES

(1) Copyediting and Proofreading: The corresponding author will receive a typeset proof for review, to ensure errors have not been introduced during production. Please review the PDF proof of your manuscript carefully, as this is the last chance to correct any errors. Please note that major changes, or those which affect the scientific understanding of the work, will likely cause delays to the publication date of your manuscript.

(2) Appropriate Figure Files: Please remove all name and figure # text from your figure files. Please also take this time to check that your figures are of high resolution, which will improve the readbility of your figures and help expedite your manuscript's publication. Please note that figures must have been originally created at 300dpi or higher. Do not manually increase the resolution of your files. For instructions on how to properly obtain high quality images, please review our Figure Guidelines, with examples at: http://journals.plos.org/plospathogens/s/figures.

We encourage you to use PACE (http://pace.apexcovantage.com/), a figure-checking tool which helps authors ensure all figures meet PLOS requirements so that figures can publish in the highest quality. PACE is a digital diagnostic and conversion tool for figure files. It will provide information about any failed check(s) and, if able, will automatically convert the figure into an acceptable file that passes quality checks.

To use PACE, you must first register as a user. Then, login and navigate to the UPLOAD tab, where you will find detailed instructions on how to use the tool.

(3) Striking Image: Please upload a striking still image to accompany your article if one is available (you can include a new image or an existing one from within your manuscript). Should your paper be accepted, this image will be considered for our monthly issue image and may also appear on our website to feature your article. Please upload this as a separate file, selecting "striking image" as the file type upon upload. Please also include a separate "Other" file with a caption, including credits and any potential copyright information. Please do not include the caption in the main article file. If your image is from someone other than yourself, please ensure that the artist has read and agreed to the terms and conditions of the Creative Commons Attribution License at http://journals.plos.org/plospathogens/s/content-license. Please note that PLOS cannot publish copyrighted images.

(4) Press Release or Related Media: If you or your institution will be preparing press materials for this manuscript, please inform our press team in advance at plospathogens@plos.org as soon as possible. We ask that you contact us within one week to plan ahead of our fast Production schedule. If you need to know your paper's publication date for related media purposes, you must coordinate with our press team, and your manuscript will remain under a strict press embargo until the publication date and time.

(5) Update your Profile Information: Now that your manuscript has been provisionally accepted, please log into Editorial Manager and update your profile, if needed. Go to http://www.editorialmanager.com/ppathogens, log in, and click on the "Update My Information" link at the top of the page. Please update your user information to ensure an efficient production and billing process.

(6) LaTeX users only: Our staff will ask you to upload a TEX file in addition to the PDF before the paper can be sent to typesetting, so please carefully review our Latex Guidelines http://journals.plos.org/plospathogens/s/latex in the meantime.

Thank you again for supporting Open Access publishing; we are looking forward to publishing your work in PLOS Pathogens.

Best regards,

Martin Craig Taylor

Guest Editor

PLOS Pathogens

Dominique Soldati-Favre

Editor-in-Chief

PLOS Pathogens

***********************************************************

The revised version of the manuscript has addressed the concerns of the reviewers and is now acceptable for publication. The fourth reviewer has raised some minor points which you can address prior to publication.

Reviewer's Responses to Questions

**Part I - Summary**

Please use this section to discuss strengths/weaknesses of study, novelty/significance, general execution and scholarship.<br/><br/>

Reviewer #1: As initial review

Reviewer #3: I believe the authors have amended the manuscript or appropriately rebutted reviewers comments in a satisfactory manner

Reviewer #4: As a new reviewer brought in following the submission of the revised manuscript, I focused my assessment primarily on the authors' responses to the comments raised during the first round of review. Within my area of expertise, I find that the authors have adequately addressed the reviewers' concerns, provided well-reasoned explanations, and made the necessary revisions to the manuscript.

--------------------

**Part II – Major Issues: Key Experiments Required for Acceptance**

Please use this section to detail the key new experiments or modifications of existing experiments that should be absolutely required to validate study conclusions.<br/><br/>Generally, there should be no more than 3 such required experiments or major modifications for a "Major Revision" recommendation. If more than 3 experiments are necessary to validate the study conclusions, then you are encouraged to recommend "Reject".<br/><br/>

Reviewer #1: N/A

Reviewer #3: I consider the experimental evidence to support conclusions (or that the proposed solutions to still ambiguous outcomes are good enough for consideration - and further debate/investigation later)

Reviewer #4: (No Response)

--------------------

**Part III – Minor Issues: Editorial and Data Presentation Modifications**

Please use this section for editorial suggestions as well as relatively minor modifications of existing data that would enhance clarity.<br/><br/>

Reviewer #1: Authors have addressed initial questions and altered manuscript where required. Manuscript is now clearer and stronger.

Reviewer #3: No additional problems noted

Reviewer #4: After reading the revised manuscript, I have a few additional minor comments and questions:

Line 69: "with adipose-resident parasites adapting their metabolism to increase sterol and lipid metabolism and β-oxidation, allowing the parasites to utilise the fatty acids available in the local tissue as a source of energy [7]."

The cited study demonstrated that fatty acids are catabolised into smaller metabolites but did not establish that this pathway contributes to ATP production. I therefore suggest that the authors revise this statement to avoid implying that fatty acid catabolism has been demonstrated to serve as an energy source.

Line 129: "We used mean bioluminescence intensity (Im) to classify tissues as being of low (Log10 Im < 5.5), medium (5.5 < Log10 Im < 7.0), or high (Log10 Im > 7.0) parasite burden."

What is the rationale for defining these three categories? Are the differences between them statistically significant? Based on the images in Figure 1B, adipose tissue, mesenteric lymph nodes (mLN), and colon appear to exhibit the highest parasite burdens. Could the authors also clarify whether the animals were perfused prior to bioluminescence measurements?

Line 266: "Alternatively, as we did not perfuse brains to remove peripheral blood there may be some contamination from bloodstream-derived mRNAs."

I am not sure I understand this argument. How would potential minor contamination with parasite-derived mRNAs affect the differential expression analysis of host genes? Could the authors clarify this point?

Line 361: Parasite RNA-seq analysis (Figure 8)

Were the animals perfused prior to tissue collection? What were the levels of parasitaemia in these mice? Given the relatively low parasite burden in the brain, could intravascular parasites contribute a substantial proportion of the parasite-derived mRNA signal? If so, how might this affect the interpretation of the parasite transcriptomic data?

--------------------

PLOS authors have the option to publish the peer review history of their article (what does this mean?). If published, this will include your full peer review and any attached files.

If you choose “no”, your identity will remain anonymous but your review may still be made public.

**Do you want your identity to be public for this peer review?**  For information about this choice, including consent withdrawal, please see our Privacy Policy.

Reviewer #1: No

Reviewer #3: No

Reviewer #4: Yes: Luisa Figueiredo

# Editor report: Acceptance letter

**DOI:** 10.1371/journal.ppat.1014734.r004

**Authors:** Tracey Lamb, Martin Taylor

Dear Dr. Urbaniak,

We are delighted to inform you that your manuscript, "Adaptive metabolic reprogramming of the brain tissue during late-stage Trypanosoma brucei infection maintains host learning and memory," has been formally accepted for publication in PLOS Pathogens.

We have now passed your article onto the PLOS Production Department who will complete the rest of the pre-publication process. All authors will receive a confirmation email upon publication.

Thank you again for supporting open-access publishing; we are looking forward to publishing your work in PLOS Pathogens.

Best regards,

Dominique Soldati-Favre

Editor-in-Chief

PLOS Pathogens
